# Editorial: The insights of multi-omics into the microenvironment after tumor metastasis: a paradigm shift in molecular targeting modeling and immunotherapy for advanced cancer patients, volume II

**DOI:** 10.3389/fonc.2026.1986038

**Published:** 2026-10-22

**Authors:** Mengyan Xie, Hong Chang, Xue Zhao, Jie Huang, Hailin Tang, Divya Gopinath, Xinhua Xiao, Chao Wang

**Affiliations:** 1 Department of Oncology, Shanghai Jiaotong University School of Medicine Affiliated Ruijin Hospital, Shanghai, China; 2 Ruijin-XJTLU Intelligent Medicine Institute, Ruijin Hospital, Shanghai Jiao Tong University School of Medicine, Shanghai and Xi’an Jiaotong-Liverpool University, Suzhou, China; 3 Xi’an Jiaotong-Liverpool University, Suzhou, China; 4 The Second Department of Internal Medicine, Shanghai Corps Hospital of Chinese People’s Armed Police, Shanghai, China; 5 Department of General Practice, Changshou Community Health Service Center of Putuo District, Shanghai, China; 6 State Key Laboratory of Oncology in South China, Guangdong Provincial Clinical Research Center for Cancer, Sun Yat-sen University Cancer Center, Guangzhou, China; 7 Basic Medical and Dental Sciences Department, College of Dentistry, Ajman University, Ajman, United Arab Emirates; 8 Centre of Medical and Bio-allied Health Sciences Research, Ajman University, Ajman, United Arab Emirates; 9 Department of Hematology and Oncology, Women and Children’s Medical Center, Guangzhou Medical University, Guangzhou, China

**Keywords:** advanced cancer, immunotherapy, metastasis, microenvironment, multi-omics

Metastasis remains the primary cause of death in cancer patients (1, 2). Even with new targeted drugs and immune checkpoint blockers (3), many patients with advanced disease do not achieve long-term remission (4). The tumor microenvironment after metastasis is an active ecosystem where cancer cells constantly change their surroundings to grow, evade immunity, and resist treatment (3). Traditional bulk-omics average signals across many cell types, masking rare but clinically decisive subclones (5). The second volume of our Research Topic addresses this gap through single-cell transcriptomics, spatial profiling, proteomics, epigenomics, radiomics, and functional validation. The 15 articles collected here converge on a central tenet: effective therapy for advanced cancer must target both the seed and the soil, and multi-omics provides the roadmap.

Several studies identify molecular hubs operating at the tumor-stroma interface. Yi et al. present a comprehensive multi-omics review demonstrating that SERPINE2 is consistently overexpressed in metastatic tumors. Mechanistically, SERPINE2 stabilizes EGFR by blocking c-Cbl-mediated ubiquitination, sustaining STAT3 and ERK signaling, while enhancing ataxia telangiectasia mutated (ATM)-driven DNA repair and radioresistance. As a secreted factor, it polarizes macrophages toward M2, activates cancer-associated fibroblasts (CAFs), and remodels extracellular matrix (ECM). This dual tumor-intrinsic and stromal function positions SERPINE2 as a molecular hub in post-metastatic adaptation.

Metabolic reprogramming emerges as a central driver. Jiang et al. show that TRIM25, an E3 ligase overexpressed in hepatocellular carcinoma, suppresses ferroptosis by reducing ferrous iron and lipid peroxidation while boosting antioxidant defenses. Its knockdown sensitizes cells to ferroptotic death and correlates with worse survival in The Cancer Genome Atlas (TCGA) and International Cancer Genome Consortium (ICGC) cohorts. Extending metabolism to hematological malignancies, Liu et al. identify lysosomal alpha-glucosidase (GAA), a lysosomal enzyme for glycogen degradation, as upregulated in angioimmunoblastic T-cell lymphoma patients with poor chemotherapy response. GAA-high tumors show enhanced glycolysis, reduced oxidative phosphorylation, and CD8^+^ T-cell exhaustion signatures, validated in independent Gene Expression Omnibus (GEO) cohorts. These findings point to glycogen metabolism as a shared vulnerability across solid and liquid tumors.

Innate immunity and epigenetics converge in a neutrophil extracellular trap (NET)-driven axis. Cai et al. show that IL-17A-bearing neutrophil extracellular traps promote H2BC4 acetylation via p300 recruitment, driving epithelial-mesenchymal transition (EMT) and brain metastasis in lung adenocarcinoma. Disrupting neutrophil extracellular traps (NETs) with PAD4 inhibitors or IL-17A neutralization reduced metastasis in mouse models.

Cell-type specificity is illuminated by single-cell and spatial atlases. Li et al. demonstrate that PTPRR, almost exclusively expressed in pancreatic ductal epithelial cells, is elevated in metastatic lesions and correlates with poor survival. PTPRR-high cells form niches with inflammatory CAFs via endothelin (EDN), EGF, and PDGF signaling, and engage TGF-β, CCL, and CXCL to foster immunosuppression.

In papillary thyroid carcinoma, Chen et al. reveal that papillary thyroid carcinoma (PTC) without thyroiditis exhibits ECM remodeling and PI16^+^ fibroblast activation, whereas PTC with thyroiditis involves immune pathways without fibroblast infiltration. A 17-gene machine-learning model, with SHISA5 identified by Mendelian randomization as a causal gene, effectively predicts lymph node metastasis.

Several studies deliver clinically actionable tools. Cui et al. construct a five-gene esophageal squamous cell carcinoma (ESCC) model (BH3 interacting domain death agonist (BID), CBX3, ECT2, KIF14, SORBS2) reaching area under the curve (AUC) 0.99, with SORBS2 as a protective factor in myofibroblasts. In gastric cancer, Hu et al. identify RPS29, an ECM-related ribosomal protein, as a hub promoting migration and EMT; its high expression predicts sensitivity to trifluoperazine.

For immunotherapy response, Xie et al. develop a pan-cancer model based on dynamic CD8^+^ T cell decline. Combined with neutrophil-to-lymphocyte ratio (NLR), therapy line, and cancer type, it achieved time-dependent AUC 0.727, 0.827 and 0.853 at 2.5, 3.5 and 5.7 months, respectively. The risk score based on multivariate Cox analysis and the simplified clinical scoring system effectively stratified different prognostic groups. This is the first study to demonstrate the prognostic value of the dynamic changes in lymphocyte subsets, particularly CD8^+^ T cells, in the immunotherapy of various cancers. The pioneering risk stratification system provides a valuable theoretical basis for clinicians to make decisions regarding immunotherapy.

Another gastric cancer model from Zhou et al. using body composition parameters (muscle attenuation, NLR, weight loss) also predicted chemotherapy-plus-PD-1 response (AUC 0.728), showing the value of accessible peripheral parameters when molecular biomarkers are unavailable.

Molecular imaging advances include a novel 99mTc-FAPI-YQ3 single-photon emission computed tomography/computed tomography (SPECT/CT) study in advanced differentiated thyroid carcinoma (DTC) by Zhou et al. Dual-tracer comparison with fluorodeoxyglucose (FDG) positron emission tomography/computed tomography (PET/CT) showed only 52.4% concordance, with FDG-positive/FAPI-negative (42.7%) and FDG-negative/FAPI-positive (4.9%) lesions reflecting distinct metabolic versus stromal phenotypes. A radiomics model achieved AUC 0.951. The authors rightly caution that fibroblast activation protein inhibitor (FAPI) uptake is an imaging phenotype, not direct fibroblast activation protein (FAP) expression proof. Complementing this, Huang et al. and Huang et al. develop a computed tomography (CT)-based radiomics score (Rad-score) for non-small cell lung cancer (NSCLC) correlating with hypoxia, EMT, and immune exclusion, bridging imaging to tumor biology.

Two reviews provide macro-level integration. Li et al. synthesize evidence on lactate accumulation, lipid metabolic rewiring, ferroptosis, cuproptosis, and the tryptophan-IDO1-kynurenine axis as drivers of immune suppression, emphasizing multi-omics as essential tools for decoding these interactions. Yuan et al. perform a pan-cancer analysis of KLF5, showing it is upregulated in most cancers and associated with poor prognosis, and its knockdown suppressed proliferation and migration in glioblastoma and bladder cancer. This exemplifies how pan-cancer analyses identify shared vulnerabilities across tumor types and reveal tissue-specific functional heterogeneity.

Despite these advances, we must acknowledge the limitations that still pervade the field. First, much of the mechanistic evidence remains correlative or derived from preclinical models. Large-scale prospective validation in independent patient cohorts is scarce. The functional context-dependence of many regulators means that a one-size-fits-all therapeutic approach is unlikely to succeed. For example, SERPINE2 may act differently in lung versus liver metastases. PTPRR’s role may vary by KRAS mutation status. Second, the development of specific, clinically druggable inhibitors for these targets is still in its infancy. For extracellular targets like SERPINE2 and PTPRR, neutralizing antibodies or ligand-traps are conceptually feasible. But their pharmacokinetics, tissue penetration, and on-target toxicity in normal stroma remain unknown. Third, the spatiotemporal heterogeneity of metastatic lesions demands not only static biomarkers but also dynamic monitoring tools. Radiomics and liquid biopsies could play a pivotal role here. Fourth, while the FAPI imaging study by Zhou et al. is a pioneering effort, the small sample size (n=17) and the lack of immunohistochemical validation of FAP expression mean that the findings should be interpreted as hypothesis-generating rather than definitive. Fifth, for the GAA study in angioimmunoblastic T-cell lymphoma (AITL), the relatively small sample size and the correlative nature of the analyses preclude definitive conclusions regarding causality; functional studies manipulating GAA expression will be required to clarify its mechanistic role. Sixth, the CD8^+^ T cell dynamics model by Xie and colleagues requires external validation in multicenter prospective cohorts, and its applicability to emerging immunotherapy scenarios such as neoadjuvant therapy or adjuvant treatment without measurable lesions remains to be established.

Looking ahead, we see three priorities. First, rigorous clinical validation of multi-omics-derived signatures in multicenter, prospective trials is essential (6–8). Second, integrating spatial omics with longitudinal imaging will allow us to track TME evolution during therapy and adapt regimens accordingly. This approach aligns with the emerging concept of adaptive precision oncology (9). Third, artificial intelligence-driven multi-omics integration should move beyond cluster discovery to generate actionable predictive models that can be deployed at the bedside (4, 6). We are convinced that the insights gathered in this Research Topic will catalyze a fundamental reorientation: from treating cancer as a genetic disease to manage it as an ecosystem disorder. This shift promises to improve outcomes for patients with advanced malignancies.
